# Glucocorticoid Exposure of Preimplantation Embryos Increases Offspring Anxiety-Like Behavior by Upregulating miR-211-5p *via* Trpm1 Demethylation

**DOI:** 10.3389/fcell.2022.874374

**Published:** 2022-04-01

**Authors:** Hong-Jie Yuan, Xiao Han, Guo-Liang Wang, Jia-Shun Wu, Nan He, Jie Zhang, Qiao-Qiao Kong, Shuai Gong, Ming-Jiu Luo, Jing-He Tan

**Affiliations:** Shandong Provincial Key Laboratory of Animal Biotechnology and Disease Control and Prevention, College of Animal Science and Veterinary Medicine, Shandong Agricultural University, Tai’an City, China

**Keywords:** anxiety, glucocorticoids, miR-211-5p, preimplantation embryos, Trpm1

## Abstract

Most studies on mechanisms by which prenatal stress affects offspring behavior were conducted during late pregnancy using *in vivo* models; studies on the effect of preimplantation stress are rare. *In vivo* models do not allow accurate specification of the roles of different hormones and cells within the complicated living organism, and cannot verify whether hormones act directly on embryos or indirectly to alter progeny behavior. Furthermore, the number of anxiety-related miRNAs identified are limited. This study showed that both mouse embryculture with corticosterone (ECC) and maternal preimplantation restraint stress (PIRS) increased anxiety-like behavior (ALB) while decreasing hippocampal expression of glucocorticoid receptor (GR) and brain-derived neurotrophic factor (BDNF) in offspring. ECC/PIRS downregulated GR and BDNF expression by increasing miR-211-5p expression via promoter demethylation of its host gene Trpm1, and this epigenetic cell fate determination was exclusively perpetuated during development into mature hippocampus. Transfection with miR-211-5p mimic/inhibitor in cultured hippocampal cell lines confirmed that miR-211-5p downregulated Gr and Bdnf. Intrahippocampal injection of miR-211-5p agomir/antagomir validated that miR-211-5p dose-dependently increased ALB while decreasing hippocampal GR/BDNF expression. In conclusion, preimplantation exposure to glucocorticoids increased ALB by upregulating miR-211-5p via Trpm1 demethylation, and miR-211-5p may be used as therapeutic targets and biomarkers for anxiety-related diseases.

## Introduction

Both epidemiological studies in humans (Van den Bergh et al., 2017) and research using animal models (Weinstock, 2017) suggest that stress exposure during gestation may increase offspring’s incidence of numerous neuropsychiatric disorders including anxiety. The early stages of pregnancy are more vulnerable to stress than later stages (Glynn et al., 2001). Furthermore, 68% of women who experienced an affective disorder during pregnancy showed symptoms during the first trimester (Kitamura et al., 1993). However, most of the published studies on the effect of prenatal stress on offspring were conducted during late pregnancy; only one study was performed during the preimplantation period (Burkuš et al., 2015).

The mechanisms by which prenatal stress affects offspring behaviors are largely unknown. Studies carried out during late pregnancy revealed that stress hormones such as glucocorticoids are among the major factors responsible for the effects of maternal prenatal stress (Zagron and Weinstock, 2006; Reynolds, 2013), which are mediated, at least in part, by the maternal-placental-fetal neuroendocrine axis (Wadhwa, 2005). However, how stressors during the preimplantation period affect offspring when a maternal-placental-fetal system has not yet been established, and whether direct exposure of early embryos to hormone causes offspring behavioral alterations are unclear. Although preimplantation restraint stress (PIRS) of female mice significantly increased cortisol levels in serum and oviducts (Zheng et al., 2016), whether and how the PIRS-induced glucocorticoid elevation would affect offspring’s behavior is not known. Furthermore, preimplantation embryos can be cultured *in vitro* to specify the effect of a specific hormone on offspring behavior, which is impossible by the *in vivo* approach that has to deal with multiple hormones.

It has been proposed that microRNAs (miRNAs) could be key regulators and therapeutic targets in anxiety-related disorders. Although recent studies have revealed some anxiety-regulating miRNAs, including miR-15a, miR-17-92, miR-34, miR-101, miR-124, miR-135 and miR-155 (as reviewed by Murphy and Singewald, 2019), more should be identified to serve as therapeutic targets and biomarkers. Our predictions using PicTar, miRDB, miRanda, TargetScan, DIANA and/or miRNA.org indicated that miR-101a-3p, miR-210-3p, miR-1a-3p and miR-211-5p targeted the Gr and/or Bdnf genes. Our miRNA sequencing indicated that all these four miRNAs were upregulated in offspring from PIRS or embryo culture with corticosterone (ECC) compared with control offspring (data to be published). miR-211-5p is an intronic miRNA, with the transient receptor potential cation channel, subfamily M, member 1 (Trpm1) gene as its host gene (Mazar et al., 2010). However, our WGBS indicated that of the four miRNAs, only DNA methylation of Trpm1 promoter was decreased significantly following PIRS or ECC. Furthermore, glucocorticoid-induced loss of DNA methylation has been observed in neural progenitor cells (Bose et al., 2015).

There are many reports that the level of anxiety behavior is negatively correlated with the level of glucocorticoid receptor (GR) and brain-derived neurotrophic factor (BDNF) expression (Chen et al., 2006; Schulte-Herbrüggen et al., 2006; He et al., 2016). We therefore hypothesized that glucocorticoid exposure might induce a persistent downregulation of Gr and Bdnf expression by increasing miR-211-5p expression *via* Trpm1 demethylation, and increase anxiety-like behavior (ALB) in offspring. In this study, by using both *in vivo* and *in vitro* models, we tested whether and how direct exposure of preimplantation embryos to glucocorticoids would alter offspring’s ALB, and whether and how miR-211-5p would be involved in ALB neuropathogenesis.

## Materials and Methods

The experimental procedures used for animal care and handling were approved by the Animal Care and Use Committee of the Shandong Agricultural University, China (Approval number: SDAUA-2014-011). Unless otherwise specified, all chemicals and reagents used were purchased from Sigma Chemical Co. (St. Louis, MO, United States).

### Mice and Restraint Treatment

Mice of the Kunming strain, originally derived from ICR (CD-1), were kept in a room with a constant temperature (22–25°C) and 14 h/10 h light-dark cycles, with the dark starting at 20:00. Virgin female mice were mated at 8–9 weeks of age and checked daily at 07:30 for copulatory plugs. Presence of a copulation plug denoted gestation day 1, and the pregnant mice were paired by weight and randomly assigned to PIRS or control groups. The PIRS took place from 08:00 to 20:00 each day from day 1 to day 3 of pregnancy. Procedures for restraint and observation on food/water intake were those reported by Zhang et al. (2011). For restraint, an individual mouse was put in a micro-cage, which was placed in a large home cage. Food and water were provided during the restraint sessions. Control mice remained in their home cages when treated mice were stressed. To measure food/water intake, restrained and control mice were individually kept in cages with the floor covered by a pressboard. Food (including that crushed on the floor) and water were weighed both before and after each restraint session.

### Embryo Collection and Culture

Zygotes were collected from oviduct ampullae of unstressed mice at 14:00 on Day 1 of gestation, and were stripped of cumulus cells by pipetting in M2 medium containing 0.1% hyaluronidase. Then, zygotes were cultured for 4 days in CZB medium with or without 10^–5^ M corticosterone before embryo transfer. About 30 embryos were cultured in a 100 µL drop at 37.5°C under humidified atmosphere with 5% CO_2_ in air. Glucose (5.5 mM) was added to CZB when the embryos developed beyond 4-cell stages. Corticosterone was dissolved in absolute ethanol to prepare a 1,000× stock solution, which was stored at −20°C until use. Immediately before embryo culture, 0.1 µL of stock solution was added to 100 µL drop. To culture embryos without corticosterone, 0.1 µL of vehicle (ethanol) was added to 100 µL drop. To collect *in vivo* embryos for embryo transfer, mice that had been exposed to PIRS were sacrificed at 20:00 on day 4 of gestation and their uteri were flushed.

### Embryo Transfer

Female mice of 8–10-week-old were mated with vasectomized males, and those showing a vaginal plug the next morning were used for pseudopregnant recipients (Wu et al., 2015). Embryo transfer was performed at 20:00 on day 3 post coitus of the recipients. Fifteen good-quality blastocysts were transferred to each recipient, seven or eight per uterine horn. After the embryo transfer, the recipients were housed singly in cages until parturition.

### Observation on Pregnancy Outcome and Management of Pups Before Experiments

To observe pregnancy outcome, at the end of PIRS or after the embryo transfer, the pregnant mice or recipients were housed singly in cages until parturition. Rates of term pregnancy, litter sizes and birth weights of young were determined immediately after parturition. Male and female offspring were weaned 21 days after birth. Pups from PIRS mice were randomly culled to four males and four females per litter, and the four litter mates of the same sex were housed in one cage until use for experiments. All the pups from embryo transfer recipients were kept without culling but male and female offspring were housed separately before experiments.

### Stereotaxic Surgery and Intrahippocampal Microinjection

Mice were anesthetized with 250 mg/kg 2,2,2-tribromoethanol (Sigma-Aldrich) by intraperitoneal injection and were secured in a stereotaxic apparatus (Stoelting Co, Wood Dale, IL, United States). After being sterilized, the scalp was incised along the middle of the head to expose the skull. Then, miR-211 agomir, antagomir or scrambled negative control (Guangzhou RiboBio Co., Ltd., Guangzhou, China) were stereotaxically delivered into bilateral hippocampus (AP: 2.5 mm; ML: ±1.8 mm; DV: −1.8 mm from the Bregma). A total of 1 μL volume was delivered over 5 min to allow for full diffusion of the injected solution. After 7 days of recovery, behavioral tests and molecular work were performed the injected animals.

### Behavioral Tests

Behavioral tests were performed on offspring mice of 8 weeks old, with behavior recorded via a video camera mounted on the ceiling above the center of the test device. The camera was connected to an Any-maze video tracking motion analysis system (Stoelting, Wood Dale, IL, United States) running on a personal computer. The test room has a constant temperature of 22–25°C and white noise of 50–55 db. Before each test, mice were placed in the test room for 30 min in order to habituate them to the environment. The device was cleaned with 10% ethanol after each trial to effectively remove the scent of the previously tested animal. To start the elevated plus-maze test (EPM), a mouse was placed in the central platform, facing an open arm, and was allowed to explore the maze for 5 min. Numbers of entries and time spent in each arm over the total exploration in both open and closed arms were calculated using the Any-maze software. To start the open field test (OFT), a mouse was placed in the central area, and was allowed to explore the open field for 5 min. Then the numbers of central entries, time spent in central area and distance traveled in the open field were calculated using the Any-maze software. To perform the light dark box test (LDB), mice were placed in the light side, and were allowed to move freely between the two chambers with door open for 5 min. The total number of transitions, the latency to enter the dark chamber and the time spent in light area and distance traveled in the light chamber were recorded by the Any-maze software.

### Recovery of Blood and Organs From Adult and Fetal Animals

Collection of blood and oviducts from stressed pregnant mice was conducted immediately after their release from restraint at 20:00 and that from the offspring mice was done after a 5 min restraint stress at 15:00 at the age of 8 weeks old. Mice were sacrificed by decapitation and trunk blood (about 1 ml) was collected into ice-cooled centrifugal tubes. Hippocampus and other organs were dissected from adult mice 1 week after the behavioral test. Fetal organs were collected by caesarean sections on live pregnant mice under anesthesia on day 19 of pregnancy.

### Hormone Assay

Serum and supernatant from oviduct homogenates were prepared as previously described (Zheng et al., 2016). Corticosterone concentrations were measured using corticosterone elisa kit (Arbor Assays, K014-H1), according to the manufacturer’s instructions. The minimum level of detection was 7.7 pg/ml, and the intra- and inter-assay CVs were <6.5% and <9.9%, respectively. Cortisol levels were measured by radioimmunoassay at the Central Hospital of Tai-An City using commercial kits (Wei-Fang (3 V) Bioengineering Co. Ltd., Wei-Fang city, China). The minimum level of detection was 0.15 ng/ml, and the intra- and inter-assay CVs were 5.3 and 6.7%, respectively.

### Quantitative Real-Time PCR

Homogenization of hippocampus and other organs was performed using Trizol reagent. Approximately 50–60 embryos were collected with minimal M2 medium. The total RNA was extracted using the PicoPure RNA Isolation Kit (Applied Biosystems) with on-column DNase treatment (Qiagen), and miRNA was extracted using the miRNeasy Micro Kit (Qiagen) according to the manufacturer’s instructions. Reverse transcription for mRNA was performed using Transcriptor Reverse Transcriptase (Roche), and that for miRNA was conducted with Mir-X miRNA First-Strand Synthesis Kit (Takara). qRT-PCR for mRNAs was performed using SYBR Green (Agilent) and that for miRNA was conducted with TB Green Premix Ex Taq II (Takara) on a Mx3005P real-time PCR instrument (Stratagene, Valencia, CA) according to the manufacturer’s recommendations. Gene expression was normalized to Gapdh for mRNA and to U6 for miRNA internal control. All values were then expressed relative to calibrator samples using the 2^–(ΔΔCT)^ method. Gene-specific primers for real-time PCR are shown in Supplementary Table S1.

### Western Blotting

Western blotting was performed as previously described (Zheng et al., 2016). The primary antibodies used included rabbit anti-GR polyclonal antibody (1:1,000, ab196944; Abcam Co., Ltd.), Rabbit Anti-BDNF antibody (1:1,000, ab108319; Abcam Co., Ltd.) and mouse anti-ACTIN antibodies (1:1,000, CW0096M, CWBio Co., Ltd.), and the secondary antibodies included goat anti-rabbit IgG (1:2000, CW0111, CWBio Co., Ltd, Beijing, China) and goat anti-mouse IgG (1:2000, CW0110, CWBio Co., Ltd). Levels of GR and different BDNF forms were expressed as ratios of GR or BDNF/β-actin.

### Immunofluorescence

Embryos were incubated with rabbit anti-GR polyclonal antibody (1:1,000, ab196944; Abcam Co., Ltd.) or Rabbit Anti-BDNF antibody (1:1,000, ab108319; Abcam Co., Ltd.). The secondary antibody was Cy3-conjugated AffiniPure goat anti-rabbit IgG (1:200; 111-165-003; Jackson ImmunoResearch, West Grove, PA, United States). The relative protein content of an embryo was quantified by measuring fluorescence intensities of individual blastomeres from 4-cell embryos cultured with or without corticosterone. To ensure accuracy of quantification, we took all pictures with identical settings, and on a single plane with maximum fluorescence intensity. We measured fluorescence intensities on the raw images using Image-Pro Plus software (Media Cybernetics Inc.) under fixed thresholds across all slides. We measured both the fluorescence density and the area of the objects giving fluorescence, and calculated the mean relative intensity of fluorescence for each blastomere. We set the average relative fluorescence of embryos cultured without corticosterone to one, and expressed the averages of embryos cultured with corticosterone relative to this value.

### Cell Culture and Transfection

The mouse hippocampal neuron line HT22 was purchased from Procell (Wuhan, China). All cells were cultured in Dulbecco’s Modified Eagle’s Medium (Gibco, United States) supplemented with 10% fetal bovine serum and 1% Penicillin and Streptomycin at 37.5°C in a humidified 5% CO2 atmosphere. The miR-211-5p mimics and negative control (NC), as well as small interfering RNA (siRNA) that targeted Gr mRNAs, were synthesized by Guangzhou RiboBio (Guangzhou, China). The sense strands of targeting siRNAs for the Gr gene included siRNA-1 (5′-CCA GAG ATG TTA GCT GAAA -3′), siRNA-2 (5′-GGA TAA GTC CAT GAG TATT -3′), siRNA-3 (5′-TAG CTG AAA TCA TCA CTAA -3′) and siR-RiboTM for negative control. Briefly, 50 nM mimics or siRNAs or negative control were transfected into cells using a riboFect™ CP Transfection Kit (RiboBio). Cells were cultured in a 24-well plate before transfection. When cells grew to 30–50% of confluence, the miRNAs or siRNAs were mixed with riboFECT CP Buffer and riboFECT CP Reagent and incubated for 15 min at room temperature. The mixture was then added to each well and incubated for 48 h.

### Microinjection of Zygotes With miRNA Mimics or siRNAs

Microinjection was performed under an inverted microscope equipped with differential interference contrast. Zygotes were transferred into HCZB medium in a Petri dish, covered with mineral oil and placed on the stage of the microscope. About 10 pL of 50 µM mimic or negative control or 100 µM siRNA were injected into each zygote using a micropipette with an inner diameter of 3 µm. Immediately after injection, zygotes were cultured (about 30 embryos/100 µL drop) in CZB medium at 37.5°C under humidified atmosphere with 5% CO_2_ in air.

### Bisulfite Sequencing of the Trpm1 Gene

Our WGBS performed on the hippocampus of male offspring from ECC (date not shown) revealed a differentially methylated region (DMR) in the promoter of Trpm1 (Chr7: 64,226,560–64,226,639). Bisulfite sequencing was conducted to observe CpG methylation of this region. Genomic DNA was isolated from hippocampus and other organs using MiniBEST Universal Genomic DNA Extraction Kit (Takara). Bisulfite conversion of DNA was performed using an EpiTect Bisulfite Kit (QIAGEN) according to the manufacturer’s instructions. Bisulfite conversion of DNA from blastocysts was performed as previously described (Chen et al., 2004). The converted DNA was then amplified by nested PCR (first: forward TTA TTA AAG AAA GTT TTT TGG GGG; reverse TTC TTA CCT TTA ACT AAA AAT TCA TCATC. Second: forward TTA TTT AGT GGG ATA AAT TAT TTA AAT AG; reverse ACA ACA AAT AAT TCT AAA AAC TTC). The PCR products were purified using Gel Extraction kit (CWBiotech), and the purified PCR products were cloned into the pMD19-T Vector (Takara) and transformed into *Escherichia coli* DH5α cells (CWBiotech). At least 15 clones for each sample were sequenced using an ABI 3730xl sequencing platform (BioSune).

### Data Analysis

At least three replicates were performed for each treatment. A mixed-model regression (the Linear Mixed Models) was used to analyze data from the behavioral test. The Linear Mixed Models analyzed the effect of fixed factors (prenatal stress or corticosterone) on ALB of offspring based on the analysis of random factors (litter effect). The behavioral data were analyzed after excluding outliers deviating by more than two standard deviations from the mean. Other data, which included only one individual from a litter, were analyzed with one-way ANOVA when each measure contained more than two groups or with independent-sample t test when each measure had only two groups. A Duncan multiple comparison test was used to locate differences during ANOVA. Chi-square test was used to analyze the pregnancy rate data. The software used was Statistics Package for Social Sciences (SPSS 20, SPSS, Inc.). Data were expressed as means ± SEM, and *p* < 0.05 was considered significant.

## Results

### Embryo Culture With Corticosterone (ECC) and Embryo Transfer did not Affect Embryo Development and Pregnancy Outcome

Following embryo culture and transfer, rates of 4-cell embryos and blastocysts, and percentages of term pregnancy, live pups per pregnant recipient and birth weight of pups did not differ between embryo culture with and without corticosterone (Supplementary Table S2).

### Effects of Embryo Culture With Corticosterone on Offspring Levels of Anxiety-Like Behavior, Serum Corticosterone and Hippocampal Glucocorticoid Receptor and Brain-Derived Neurotrophic Factor

**FIGURE 1:**
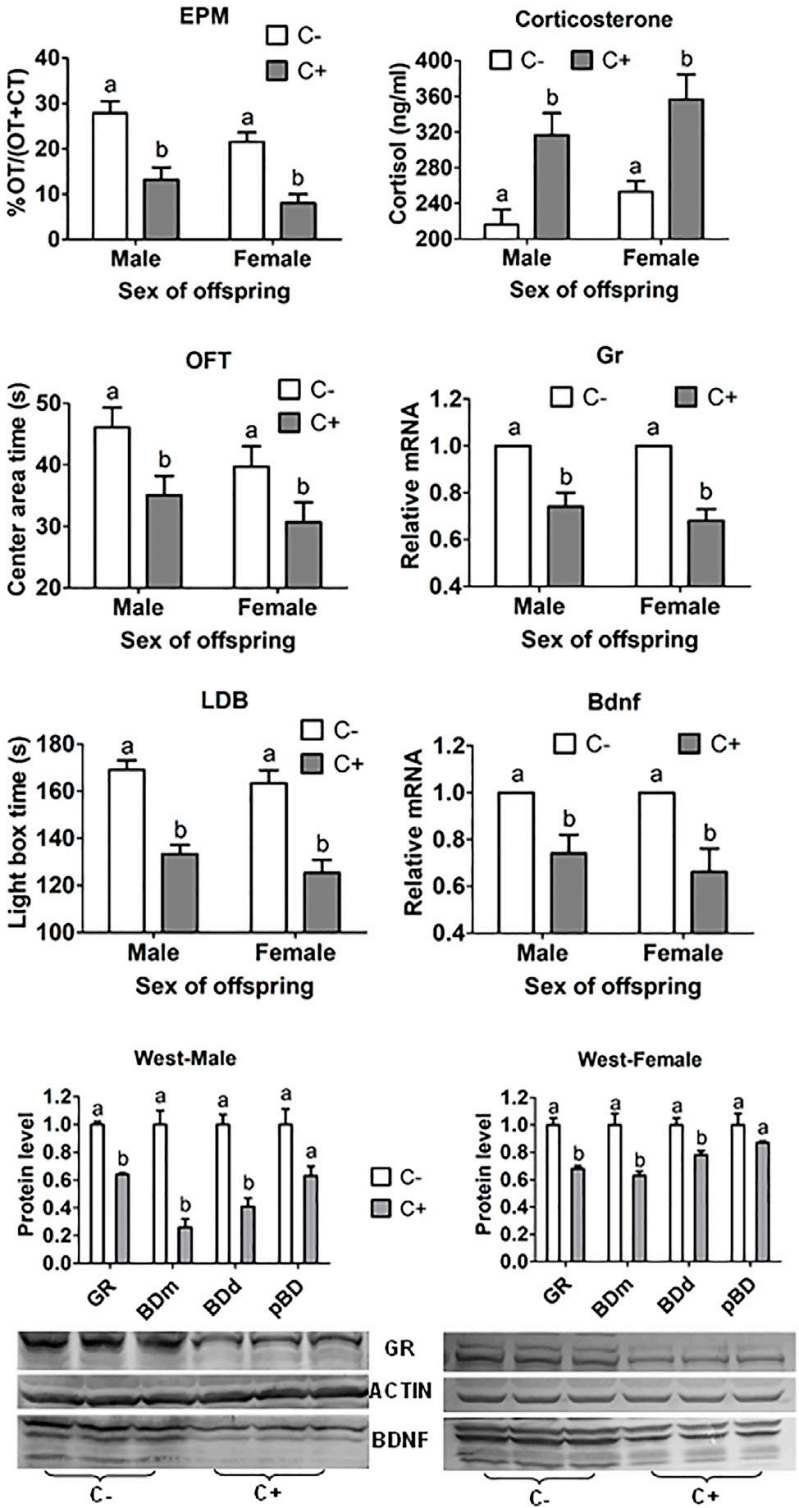
ALB, serum corticosterone concentration, and hippocampal levels of Gr and Bdnf mRNAs and protein in offspring after transfer of embryos cultured with (+) or without (−) corticosterone (C). The ALB was measured by % Open-arm time (OT)/OT + closed arm time (CT) of EPM, and times (s) in central area of OFT and in light box of the LDB test. While mRNA levels of Gr and Bdnf were measured by RT-PCR, their protein levels were measured by western blotting. For behavioral tests, each treatment was repeated three times and each replicate contained about 10 offspring from three to five recipients. For corticosterone measurement, each treatment was repeated three times with each replicate containing two to three serum samples from two to three offspring each from a different recipient. For RT-PCR and western blotting, each treatment was repeated three times with each replicate containing three hippocampus samples from three offspring each from a different recipient. Three forms of BDNF were observed including BDNF monomer (BDm), BDNF dimer (BDd) and pro-BDNF (pBD). Levels of GR and different BDNF forms were expressed as ratios of GR or BDNF/β-actin. a,b: Values with a different letter above bars differ significantly (*p* < 0.05).

In both male and female offspring from embryo transfer, whereas EPM open-arm time, OFT central area time, and LDB light box time were all shorter, serum corticosterone level was higher significantly after embryos were cultured with than without corticosterone (Figure 1). ECC also decreased hippocampal GR and BDNF mRNA and protein significantly.

### Effects of Embryo Culture With Corticosterone on Gene Expression of Preimplantation Embryos

**FIGURE 2:**
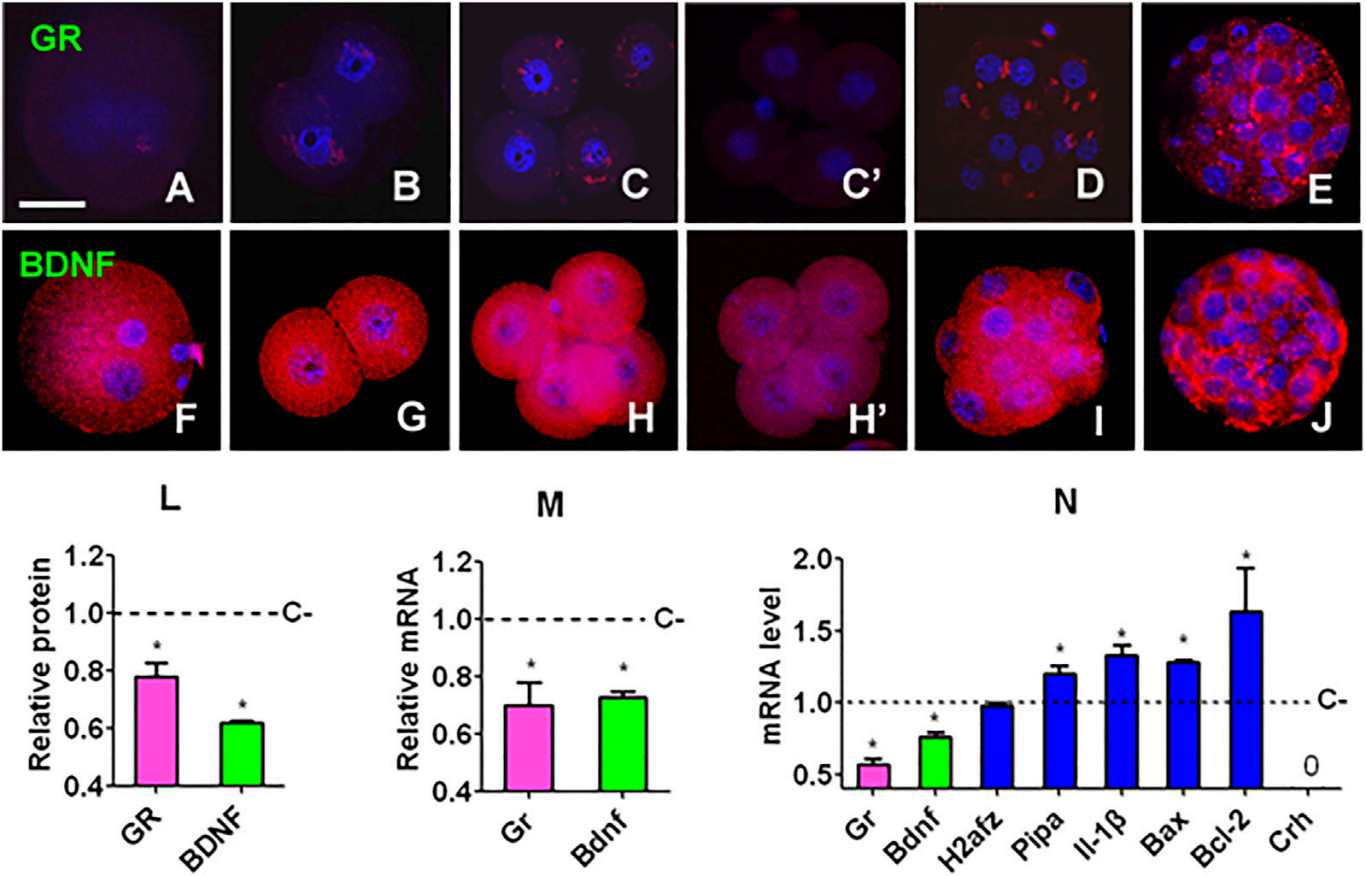
Gene expression in mouse embryos following culture with or without corticosterone. **(A,F)**, **(B,G)**, **(C,H)**, **(D,I)** and **(E,J)** Expression of GR/BDNF protein in zygote, 2-cell, 4-cell, morula and blastocyst embryos, respectively, after culture without corticosterone. **(C’,H’)** Expression of GR and BDNF, respectively, in 4-cell embryos cultured with corticosterone. The micrographs are merged images from laser confocal microscopy with GR/BDNF and DNA pseudo colored red and blue, respectively. The bar is 30 µm. **(L,M)** Relative levels of protein (immunofluorescence intensity) and mRNA (RT-PCR) of GR and BDNF, respectively, in 4-cell embryos cultured with (+) or without (−) corticosterone (C). For immunofluorescence microscopy, each treatment was repeated three times and each replicate contained 10–15 blastomeres randomly selected from 15 to 20 pooled embryos, for RT-PCR, each treatment was repeated three times with each replicate containing 60 embryos. **(N)** Relative levels of different mRNAs in blastocysts after C+ or C− culture. Each treatment was repeated three times with each replicate containing 50 blastocysts. Relative mRNA levels in C+ 4-cell embryos or blastocysts were calculated relative to those in C-embryos, which were set to 1 (dotted line). 0 indicates that the Crh mRNA was undetectable. * Indicates a significant difference (*p* < 0.05) from control (C−) embryos.

Preimplantation embryos expressed both GR and BDNF proteins at all the developmental stages examined (Figures 2A–J). While GR was localized in both cytoplasm and nucleus, BDNF located mainly in the cytoplasm of blastomeres. ECC significantly decreased GR and BDNF protein and mRNA in 4-cell embryos (Figures 2L,M). Furthermore, ECC significantly decreased levels of Gr and Bdnf mRNAs while increasing levels of Pipa, Il-1β, Bax, and Bcl2 mRNAs in blastocysts (Figure 2N). Thus, ECC specially downregulated GR and BDNF expression while increasing expression of other genes in preimplantation embryos.

### Effects of Embryo Culture With Corticosterone on Gr and Bdnf mRNA Levels in Different Organs of Adult and Fetal Offspring

**FIGURE 3:**
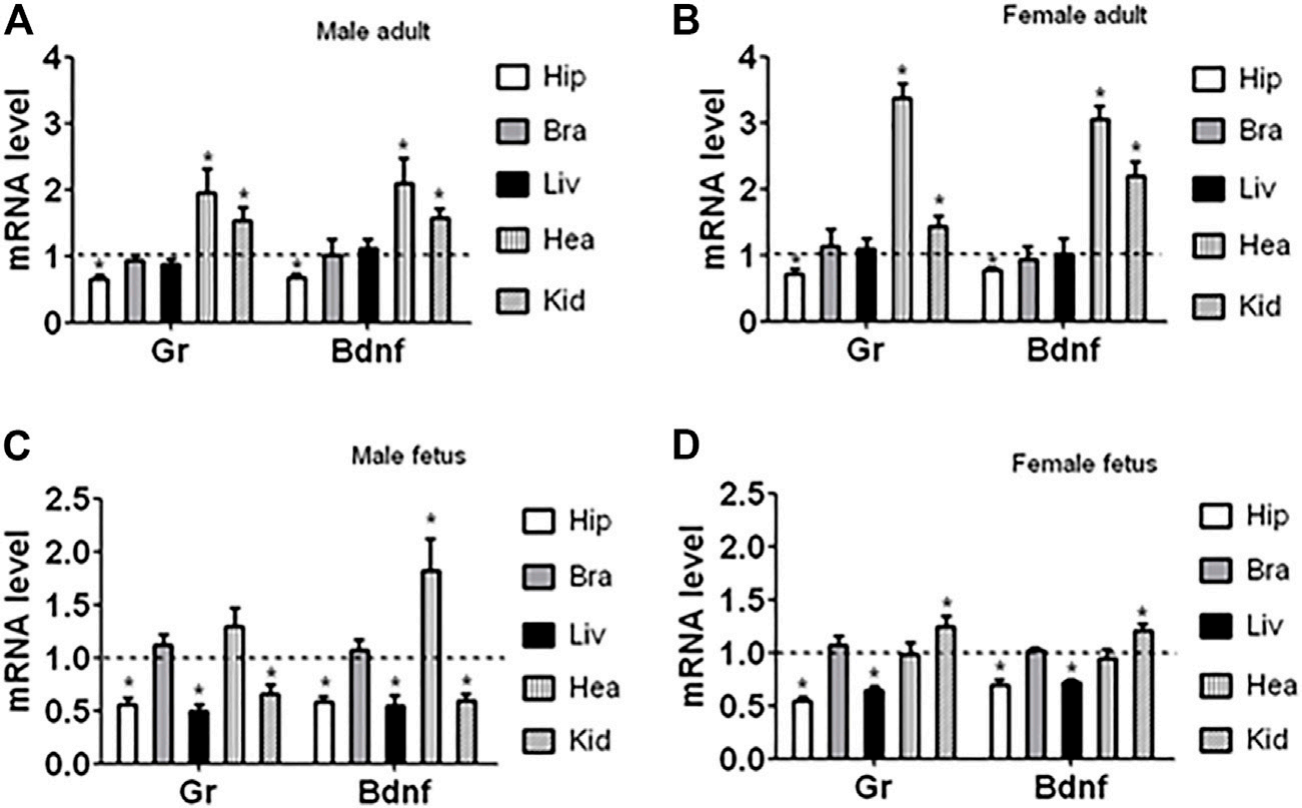
Expression of Gr and Bdnf mRNAs in different organs of adult and fetal offspring after ECC. The organs observed included hippocampus (Hip), brains with hippocampus removed (Bra), liver (Liv), heart (Hea) and kidney (Kid) from adult and fetal male and female offspring. Relative mRNA levels in the corticosterone-treated offspring were calculated relative to those in the control offspring from embryos cultured without corticosterone, which were set to 1 (dotted line). Each treatment was repeated six times with each replicate containing organs from one animal from a different litter. * Indicates a significant difference (*p* < 0.05) from offspring from embryos cultured without corticosterone.

To investigate whether the downregulation of GR and BDNF during organogenesis was hippocampus-specific and was a persistent effect initiated during preimplantation development, Gr and Bdnf mRNA levels in hippocampus, brain (after removal of the hippocampus), liver, heart and kidney of adult and fetal male and female offspring was observed following ECC. In all the samples observed, Gr and Bdnf always shared a similar trend of expression (Figure 3). Relative to control, Gr and Bdnf mRNA levels following ECC was 1) always lower in hippocampus but at the same level in the rest of the brain, 2) at the same level in adult liver but lower in fetal liver, 3) higher in heart except for the female fetal heart where it was at the same level, and 4) higher in kidney except for the male fetal kidney where it was lower. Thus, the results showed that ECC downregulated GR and BDNF expression all the way to mature hippocampus, whereas in other organs, the ECC-downregulated GR and BDNF expression was upregulated at different stages of organogenesis either to the same level or higher than that in control offspring.

### Effects of Embryo Culture With Corticosterone on Expression of miR-211-5p and Trpm1 mRNA in Blastocysts and Fetal and Adult Hippocampus of Offspring

**FIGURE 4:**
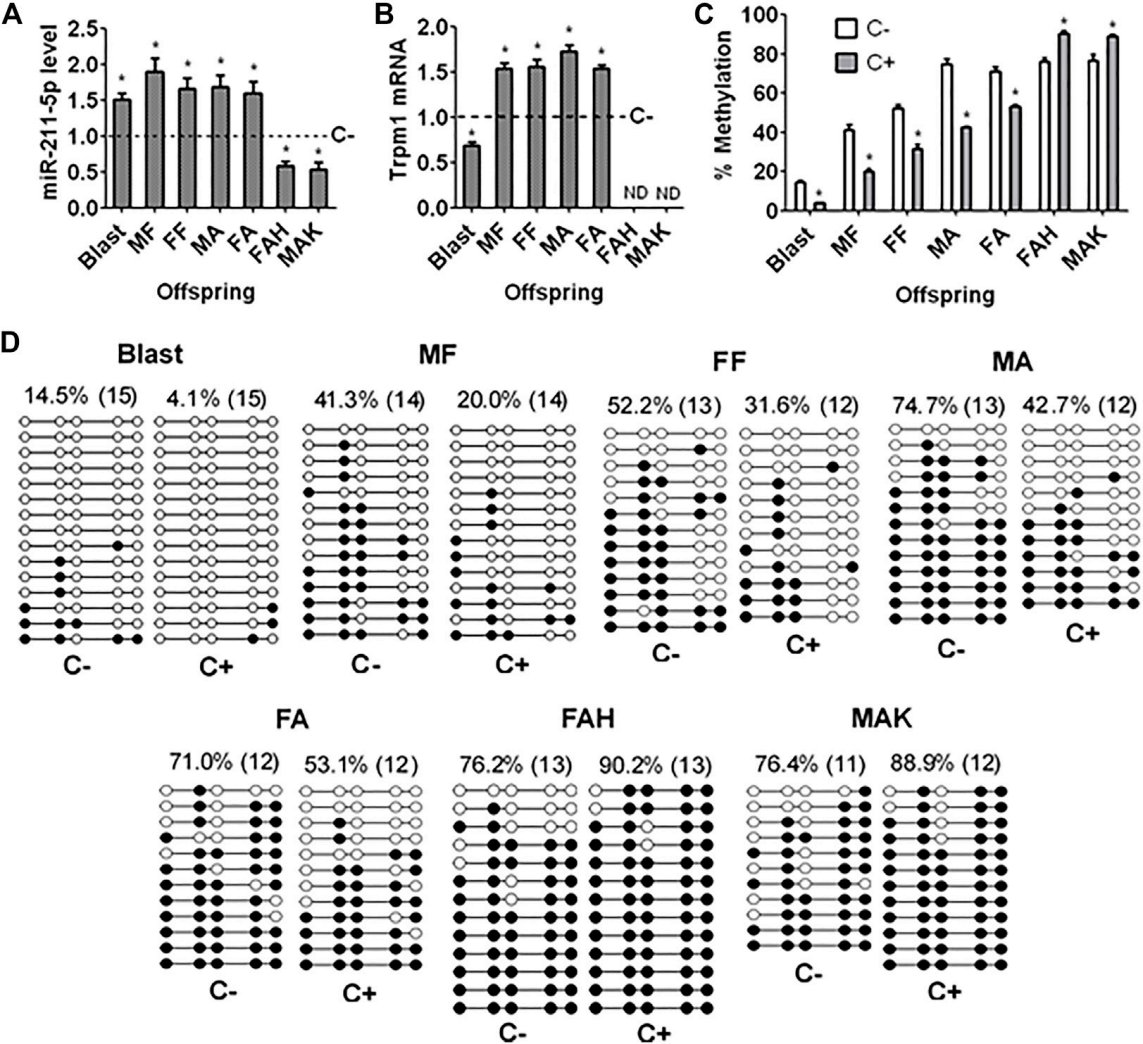
Levels of miR-211-5p and Trpm1 mRNA and methylation of Trpm1 in blastocysts and organs of offspring after ECC. **(A,B)** Levels of miR-211 and Trpm1 mRNA, respectively, as measured by quantitative real-time PCR in blastocysts (Blast), male fetal (MF), female fetal (FF), male adult (MA) and female adult (FA) hippocampus, and female adult heart (FAH) and male adult kidney (MAK) of offspring from embryos cultured with (+) or without (−) corticosterone **(C)**. Relative levels of miR-211 and Trpm1 mRNA in C+ samples were calculated relative to those in C− samples, which were set to 1 (dotted line). Trpm1 mRNA was not detectable (NB) in FAH and MAK. Each treatment was repeated 6 (graph A) or 3 (graph B) times and each replicate contained 50–60 blastocysts or organs from one fetus or adult animal from a different litter. **(C)** Percentages of methylated CpGs of the Trpm1 gene as measured by bisulfate sequencing in Blast, MF, FF, MA, FA, FAH and MAK from C+ or C− offspring. Each treatment was repeated three times with each replicate containing 30 blastocysts or organs from one animal from a different litter. * Indicates a significant difference (*p* < 0.05) from C− offspring. **(D)** DNA bisulfite sequencing of the Trpm1 gene in Blast, MF, FF, MA, FA, FAH and MAK from C+ or C− offspring. White circles indicate unmethylated CpGs, and black circles represent methylated CpGs. Values on each treatment indicate the percentage of CpG methylation, and the number in parentheses indicates clones analyzed.

miR-211-5p levels in blastocysts and the hippocampus of male and female fetal and adult offspring were significantly higher following embryo culture with than without corticosterone (Figure 4A). However, miR-211-5p expression in female adult heart and male adult kidney was significantly decreased following ECC, consistent with their trend of Gr and Bdnf expression (Figure 3). Thus, ECC downregulated Gr and Bdnf while increasing miR-211-5p expression specifically in hippocampus. Trpm1 mRNA showed a similar pattern of expression as miR-211-5p in fetal and adult hippocampus but it decreased in blastocysts following ECC (Figure 4B). Trpm1 mRNA was not detectable in female adult heart and male adult kidney.

### Effects of Embryo Culture With Corticosterone on Methylation of the Trpm1 Promoter in Blastocysts and Hippocampus of Fetal and Adult Offspring

Percentages of methylated CpGs in the promoter region of the Trpm1 gene in blastocysts and in the hippocampus of male and female fetal and adult offspring were significantly lower following embryo culture with than without corticosterone (Figures 4C,D). However, ECC significantly increased DNA methylation of Trpm1 promoter in female adult heart and male adult kidney. Thus, ECC increased miR-211 expression in blastocysts and fetal and adult hippocampus and decreased that in female adult heart and male adult kidney with down and up regulation of Trpm1 promoter methylation, respectively.

### miR-211-5p Targets Gr but Not Bdnf Expression During Preimplantation Embryo Development

**FIGURE 5:**
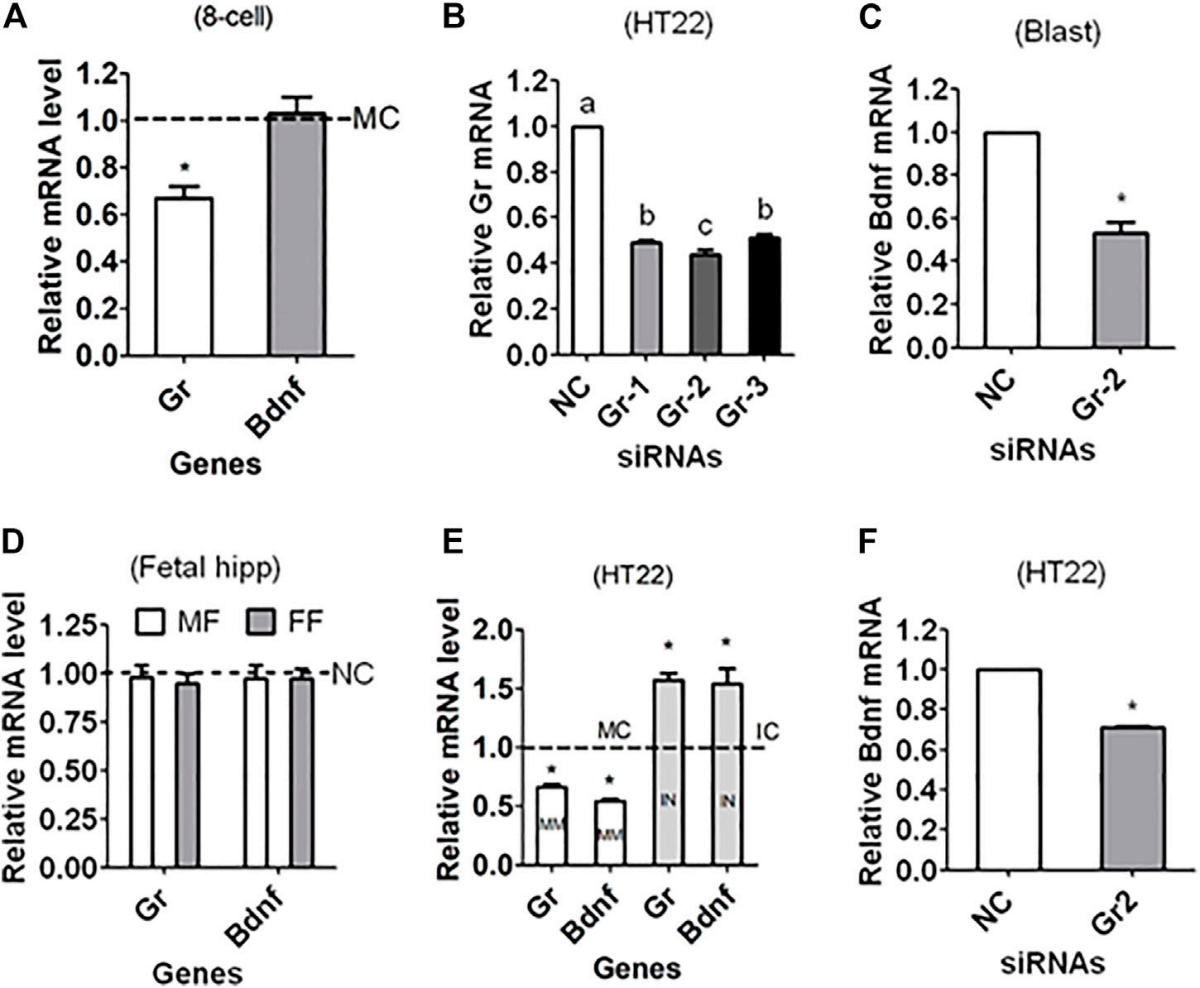
Regulating Gr and Bdnf expression in mouse preimplantation embryos and HT22 hippocampal neuron line using miR-211 mimics or Gr siRNA. **(A)** Relative levels of Gr and Bdnf mRNAs in 4- to 8-cell embryos recovered at 48 h of culture in CZB without corticosterone following microinjection of zygotes with miR-211-5p mimic negative control (MC) or mimics. **(B)** Relative levels of Gr mRNA in HT22 cell line following transfection with NC siRNA, Gr-siRNA1 (Gr-1), Gr-siRNA2 (Gr-2) or Gr-siRNA3 (Gr-3). **(C)** Relative level of Bdnf mRNA in blastocysts following microinjection of zygotes with NC or Gr-2 siRNA. **(D)** Relative levels of Gr and Bdnf mRNAs in male (MF) and female fetal (FF) hippocampus after injection of zygotes with NC siRNA or Gr-2 siRNA. **(E)** Relative levels of Gr and Bdnf mRNAs in HT22 cells following transfection with miR-211-5p mimic or inhibitor negative control (MC or IC) or mimics (MM) or inhibitors (IN). **(F)** Relative level of Bdnf mRNA in HT22 cells following transfection with NC or Gr-2 siRNA. For embryo experiments in graphs **(A,C)**, each treatment was repeated three times with each replicate containing 60 embryos; for HT22 experiments in graphs **(B,E,F)**, each treatment was repeated three times with each replicate including cells from one well of a 24-well plate; and for fetal hippocampus experiment in graph **(D)**, each treatment was repeated three times with each replicate containing one hippocampus from one offspring from a different mother. Values from NC, IC or MC transfection or injection were set to one (dotted line) and other values were expressed relative to them. * Indicates significant difference (*p* < 0.05) from the NC/MC/IC value. a–c: Values with a different letter above bars differ significantly (*p* < 0.05).

Zygotes were injected with mimic negative control (MC) or mimic of miR-211-5p before culture without corticosterone. At 48 h of the culture, 4- to 8-cell embryos were recovered for RT-PCR mRNA quantification. While the level of Gr mRNA was significantly lower in embryos injected with mimic than with MC, the level of Bdnf mRNA did not differ between mimic and MC embryos (Figure 5A).

### Effects of Downregulating Gr in Zygotes on Bdnf Expression in Blastocysts and Fetal Hippocampus

To answer the question how is Bdnf expression paralleled with Gr expression in that miR-211 targeted Gr but not Bdnf expression, RNAi targeting Gr was performed. The silencing efficiency of various siRNA sequences was first evaluated using mouse HT22 hippocampal neuronal cell line and the results showed that Gr-siRNA2 was most efficient (Figure 5B). The Bdnf mRNA level in blastocysts was significantly lower after zygotes were injected with Gr-siRNA2 than with NC siRNA (Figure 5C), confirming that GR supported BDNF expression in early embryos. When Gr and Bdnf mRNAs were measured in fetal hippocampus, however, difference was observed in neither Gr nor Bdnf mRNA between NC and Gr-siRNA injection (Figure 5D). The results showed that Gr knockdown in early embryos did not affect Bdnf mRNA expression in the hippocampus later in life, suggesting that ECC increased ALB not by downregulating Gr in early embryos but rather by other perpetuating epigenetic mechanisms.

### Regulation of Gr and Bdnf Expression by miR-211 Mimic/Inhibitor or Gr siRNA in HT22 Hippocampal Neuron Line

To verify the targeting relationship between miR-211 and Gr/Bdnf or between Gr and Bdnf in adult hippocampus, HT22 hippocampal neuron line was transfected with miR-211 negative control including mimic control (MC) or inhibitor control (IC) or with mimic or inhibitor, or with NC siRNA or Gr-siRNA2 before assessing Bdnf and/or Gr mRNAs. While both Gr and Bdnf mRNAs were lower following transfection with miR-211 mimic, both were higher after transfection with miR-211 inhibitor, compared to transfection with MC/IC (Figure 5E). The Bdnf mRNA level was significantly lower after transfection with Gr-siRNA2 than with NC siRNA (Figure 5F). The results confirmed that miR-211 downregulated Gr and Bdnf, and Gr supported Bdnf expression in adult hippocampus.

### Our Preimplantation Restraint Stress Procedures Increased Anxiety-Like Behavior and Cortisol Levels of Pregnant Mice While Affecting Neither Food/Water Intake nor Pregnancy Outcome

To further support our above results that glucocorticoid exposure of preimplantation embryos increases offspring ALB by upregulating miR-211-5p *via* Trpm1 demethylation, maternal PIRS was conducted to observe its effects on ALB and related molecular changes in offspring. Effects of PIRS on pregnant mice were first observed to test the efficiency of our PIRS procedures. When measured immediately at the end of PIRS, whereas the open arm time during EPM test was shorter, cortisol level in serum or oviduct was higher significantly in PIRS mice than in unstressed control mice (Supplementary Figure S1). Food/water intake, pregnancy rate, gestation length, litter size and birth weight of pups did not differ between PIRS and control mice (Supplementary Table S3).

### Preimplantation Restraint Stress of Pregnant Mice Increased Anxiety-Like Behavior and Serum Cortisol While Decreasing Hippocampal Gr and Bdnf mRNAs in Offspring

While the open-arm time in EPM, central area time in OFT, and light box time in LDB test were all shorter, serum cortisol level was higher significantly in both male and female offspring born to PIRS than control mothers (Supplementary Figure S2). Furthermore, PIRS significantly decreased levels of Gr and Bdnf mRNAs in hippocampus of both male and female offspring.

### Effects of Preimplantation Restraint Stress on miR-211 Expression and Trpm1 Promoter Methylation in Hippocampus of Fetal and Adult Offspring

In both male and female fetal and adult offspring, while miR-211-5p was higher, levels of Trpm1 methylation were lower significantly in PIRS than in control offspring (Supplementary Figure S3). Thus, like ECC, PIRS also increased offspring ALB through downregulating GR/BDNF by increasing miR-211-5p *via* reducing Trpm1 promoter methylation.

### Effects of Intrahippocampal Injection of miR-211-5p Agomir/Antagomir on Anxiety-Like Behavior and Expression of Related Genes

**FIGURE 6:**
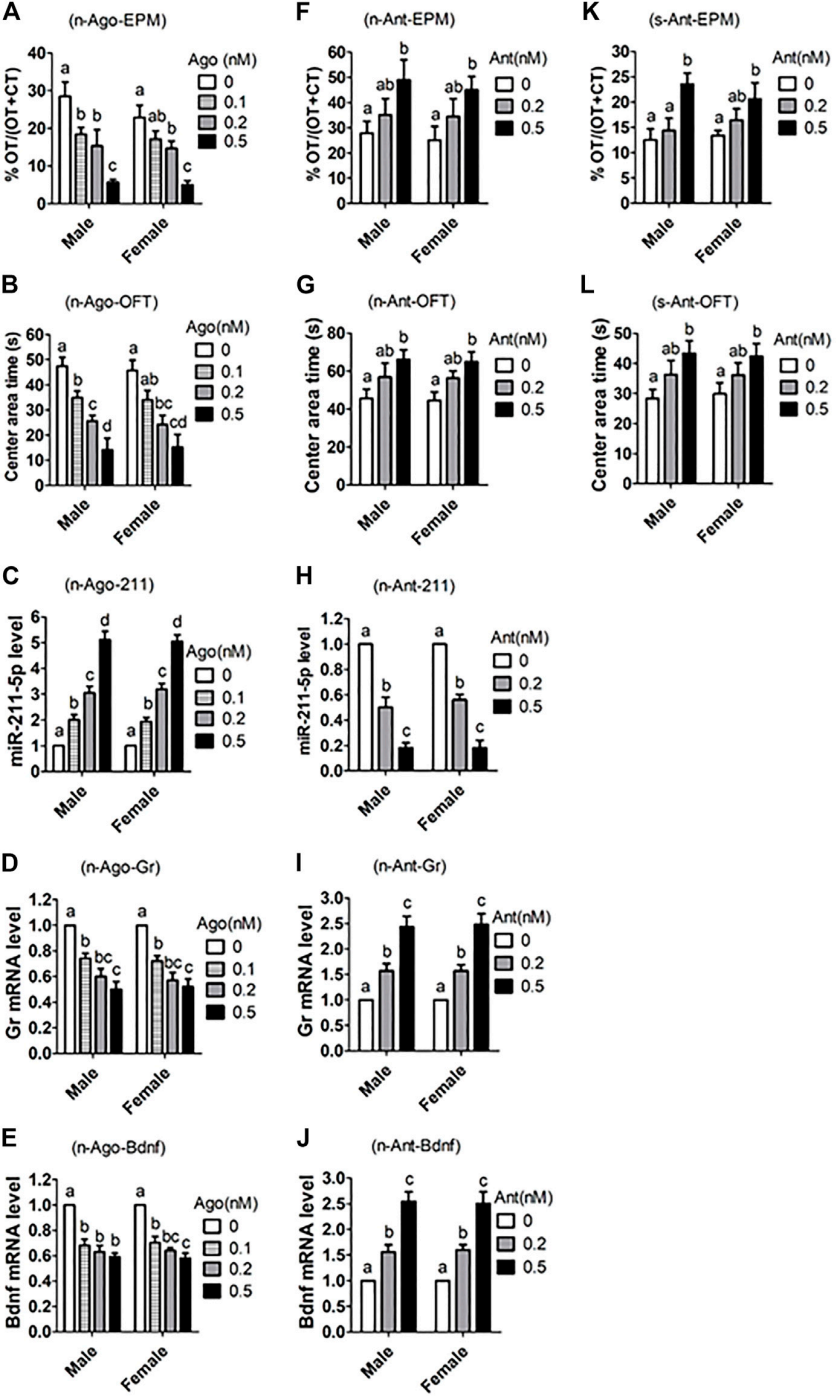
Effects of intrahippocampal injection of miR-211-5p agomir or antagomir on ALB and expression of related genes in hippocampus. Naive (n) or stressed (s) mice were intrahippocampally injected with different concentrations (nM) of miR-211 agomir (Ago) or antagomir (Ant) or scrambled negative control (0 nM). While the naive mice were randomly bred in the laboratory, the stressed mice were offspring from PIRS mothers at the same age (8–9 weeks after birth). The ALB was measured by % Open-arm time (OT)/OT + Closed arm time (CT) during EPM, and by times (s) in central area during OFT. For behavioral tests, each treatment was repeated 7 to 9 times with each replicate including one mouse, and for real-time PCR, each treatment was repeated three times with each replicate containing one mouse. **(A–E)** ALB and related parameters after normal mice were injected with miR-211 agomir, **(F–J)** ALB and related parameters after normal mice were injected with miR-211 antagomir. **(K,L)** ALB after stressed mice from PIRS mothers were injected with miR-211 antagomir. a–d: Values without a common letter above bars differ significantly within sexes.

To further verify the role of miR-211 in ALB neuropathogenesis, miR-211-5p agomir or antagomir were injected stereotaxically into the hippocampus of male and female mice before measurement for ALB and expression of related genes. In both male and female mice, whereas agomir injection dose-dependently increased ALB while decreasing Gr and Bdnf mRNA expression (Figures 6A–E), injection of antagomir decreased ALB while increasing expression of Gr and Bdnf mRNAs (Figures 6F–J). Furthermore, antagomir injection also significantly relieved ALB in PIRS offspring, which showed a much higher level of ALB than normal mice did (Figures 6K,L).

## Discussion

The present results demonstrated that ECC increased expression of miR-211-5p and Trpm1 mRNA by Trpm1 promoter demethylation in hippocampus of offspring. Glucocorticoid-induced loss of DNA methylation has been reported (Thomassin et al., 2001; Lee et al., 2010; Crudo et al., 2013; Bose et al., 2015). Recent studies showed that different treatments during culture of early embryos caused specific methylation changes at the blastocyst stage (Market-Velker et al., 2010; Barrera et al., 2017; Ashry et al., 2020). During human preimplantation development, genome-wide DNA methylation reprogramming was observed as a dynamic balance between strong global demethylation and drastic focused re-methylation (Zhu et al., 2018). Fan et al. (2016) observed that miR-211-5p impaired neurite differentiation, decreased neuronal viability and accelerated the progression of β amyloid-induced pathologies in mouse cerebral cortices. The TRPM1 protein in mouse brain has a functional relevance in modifying synaptic plasticity (Gebhardt et al., 2016). Expression of Trpm1 mRNA or protein was increased in hypothalamus of immobilization-stressed mice (Lee et al., 2005), in the transgenic Alzheimer’s disease mouse models (Yin et al., 2017), and in brain of mice showing increased ALB (Schmidt et al., 2019). However, whether the enhanced TRPM1 expression itself evoked ALB is not known. Our results confirmed that it was the increase in miR-211 promoted by Trpm1 promoter demethylation that was responsible for the increased ALB.

The current results showed that although miR-211 downregulated Gr but not Bdnf in early embryos, it downregulated both Gr and Bdnf in cultured hippocampal neuronal line and in adult hippocampus. Margue et al. (2013) demonstrated that miR-211 mimics downregulated Gr mRNA in melanoma cells. Wang et al. (2014) observed in chicken that the expression of GR mRNA and protein was associated with significantly lower expression of gga-miR-211. However, Zhang et al. (2017) reported that miR-211 inhibited BDNF expression in human astrocytes through direct targeting. The discrepancy might have resulted from species differences, as miRNA regulation can be modulated by conservation levels of their genes and binding sites and by the expression levels of themselves, which vary spatially and temporally in different species (Mor and Shomron, 2013). The functional targets of a miRNA may vary in different cells or even in different states of the same cell (Li et al., 2013). Furthermore, the strength of miRNA regulation may differ with cell lines (Kulkarni et al., 2016).

In all the tissues we examined, including early embryos and various fetal and adult organs, GR and BDNF shared similar trends of expression. The expression of Gr and Bdnf mRNAs in hippocampus was always closely correlated whether mice showed increased or decreased ALB (He et al., 2016). Expression of GR and BDNF decreased simultaneously in oviduct cells following PIRS or *in vitro* treatment with corticosterone (Zheng et al., 2016; Tan et al., 2017). Our RNAi demonstrated that Gr was essential for Bdnf transcription in preimplantation embryos and HT22 hippocampal neuron line. Ridder et al. (2005) observed a BDNF downregulation in the hippocampus of GR+/− mice with a 50% Gr gene dose reduction. Mice over-expressing GR were less susceptible to stress and displayed enhanced BDNF expression in hippocampus (Schulte-Herbrüggen et al., 2016). Furthermore, GR knockdown by intracerebroventricular administration of siRNAs significantly suppressed BDNF expression in mouse brain (Li et al., 2018).

The present results indicated that preimplantation glucocorticoid exposure downregulated GR and BDNF expression by increasing miR-211-5p expression *via* Trpm1 demethylation, and this epigenetic cell fate determination in early embryos was exclusively perpetuated during development into mature hippocampus to increase offspring’s ALB. Although it is known that during individual development, cell fate is determined by the establishment of cell-type-specific transcriptional programs through epigenetic mechanisms (Seah and Messerschmidt, 2018), how the epigenetic mechanisms guide, reinforce and ultimately lock-in the transcriptional programs in a specific cell type is largely unknown. Furthermore, published studies reported environmental cell fate determination during organogenesis not in early embryos (Ferguson and Joanen, 1982; Martins and Pearson, 2008). Thus, this is the first report that an epigenetic decision made by environmental cues at the cleavage stage is maintained into a functionally mature organ to alter offspring’s phenotype.

Our transfection with miR-211-5p mimic/inhibitor in cultured hippocampal cell lines showed that miR-211-5p decreased hippocampal Gr and Bdnf expression, and our intrahippocampal injection of miR-211-5p agomir/antagomir demonstrated that miR-211-5p dose-dependently increased ALB with decreased hippocampal Gr and Bdnf expression. While associative studies identified about 30 anxiety-related miRNAs, functional studies validated only about 10 (Murphy and Singewald, 2019; Narayanan and Schratt, 2020). Furthermore, miR-211-5p was not in the list of the identified anxiety-regulating miRNAs. Thus, our study has demonstrated for the first time that miR-211-5p plays a pivotal role in ALB pathogenesis and it may be used as therapeutic targets and biomarkers for human anxiety-related disorders.

In conclusion, this study demonstrated for the first time that *in vitro* or *in vivo* exposure of preimplantation embryos to glucocorticoids upregulated miR-211-5p expression by Trpm1 demethylation; the increased miR-211-5p in turn downregulated Gr and Bdnf expression; and this epigenetic cell fate determination was exclusively perpetuated during development into mature hippocampus to increase ALB in offspring. Thus, the results have revealed the mechanisms by which preimplantation glucocorticoid exposure increases offspring’s ALB and the pivotal role for miR-211-5p in ALB neuropathogenesis, suggesting that miR-211-5p may be used as therapeutic targets and biomarkers for human anxiety-related diseases.

## Data Availability

The original contributions presented in the study are included in the article/Supplementary Material, further inquiries can be directed to the corresponding author.
