# Supplementary material for: Glucocorticoid Exposure of Preimplantation Embryos Increases Offspring Anxiety-Like Behavior by Upregulating miR-211-5p via Trpm1 Demethylation
Source: Front Cell Dev Biol. 2022 Apr 1;10:874374. doi: 10.3389/fcell.2022.874374 (PMC9011152; doi:10.3389/fcell.2022.874374)
Supplement: Supplementary file 1 [file Table1.DOCX]

**Supplementary tables and figures**

Title: Glucocorticoid exposure of preimplantation embryos increases offspring anxiety-like behavior by upregulating miR-211-5p via Trpm1 demethylation

Running title: Glucocorticoid, early embryo, miRNA-211 and anxiety

Hong-Jie Yuan^1^, Xiao Han^1^, Guo-Liang Wang^1^, Jia-Shun Wu^1^, Nan He^1^, Jie Zhang^1^, Qiao-Qiao Kong^1^, Shuai Gong^1^, Ming-Jiu Luo^1^ and Jing-He Tan^1,2^

^1^ Shandong Provincial Key Laboratory of Animal Biotechnology and Disease Control and Prevention, College of Animal Science and Veterinary Medicine, Shandong Agricultural University, Tai'an City 271018, P. R. China

^2^ Corresponding author: Jing-He Tan, College of Animal Science and Veterinary Medicine, Shandong Agricultural University, Tai-an City, Shandong Province, P R China, Post code: 271018, Phone: 0538-8249616, FAX: 0538-8241419, Email: [tanjh@sdau.edu.cn](mailto:tanjh@sdau.edu.cn)

Table S1. Gene-specific primers used for real-time PCR

| Genes | Primers (5’-3’) |
| --- | --- |
| *Gr* | F: AGTCAAGGTTTCTGCGT  R: CCATCACTTTTGTTTCG |
| *Bdnf* | F: GCCTCCTCTACTCTTTCTG  R: GGATTACACTTGGTCTCGT |
| *Gapdh* | F: AAGGTGGTGAAGCAGGCAT  R: GGTCCAGGGTTTCTTACTCCT |
| *Ppia* | F: CGCGTCTCCTTCGAGCTGTTTG  R: TGTAAAGTCACCACCCTGGCACAT |
| *H2afz* | F: ACAGCGCAGCCATCCTGGAGTA  R: TTCCCGATCAGCGATTTGTGGA |
| *Crh* | F: CCTCAGCCGGTTCTGATCC  R: GCGGAAAAAGTTAGCCGCAG |
| *IL1β* | F: GAAATGCCACCTTTTGACAGTG  R: TGGATGCTCTCATCAGGACAG |
| *Bcl2* | F: TTCGGGATGGAGTAAACTGG  R: TGGATCCAAGGCTCTAGGTG |
| *Bax* | F: TGCAGAGGATGATTGCTGAC  R: GATCAGCTCGGGCACTTTAG |
| *Trpm1* | F: CTCCCGAAGCTCTTGATATCTG  R: AGCCTTGATCAGACCTTTCC |
| miR-211-5p | F: TTCCCTTTGTCATCCTTTGCCT  R: mRQ 3’ Primer (supplied by Takara kit) |
| U6 | F: TGGAACGCTTCACGAATTTGCG  R: GGAACGATACAGAGAAGATTAGC |

Table S2. Effects of culture with (+) or without (-) corticosterone (C) on embryo development in vitro and in vivo after embryo transfer

| Embryo treatment | In vitro culture | | After embryo transfer | | |
| --- | --- | --- | --- | --- | --- |
|  | % 4-cell embryos | % Blastocysts | % Term pregnancy of recipients | Live pups per pregnant recipient | Birth weight of pups (g) |
| C- | 96.9±1.1 | 85.8±4.8 | 80.0 | 7.8±1.0 | 1.96±0.05 |
| C+ | 96.1±1.0 | 85.4±3.5 | 81.3 | 6.9±0.8 | 2.05±0.03 |
| PIRS | NA | NA | 78.6 | 7.0±0.3 | 2.01±0.02 |

All the values did not differ significantly (P > 0.05) within columns. For in vitro culture, each treatment was repeated 3-4 times with each replicate containing 50-60 zygotes. In the embryo transfer experiment, each treatment was repeated 3 times with each replicate containing 5 recipients. NA: Not applicable.

Table S3. Food/water intake and pregnancy outcome after PIRS of pregnant mice

| Mouse treatment | Food (g) | Water (ml) | Pregnancy rate (%) | Gestational period (d) | Litter size | Birth weight (g) |
| --- | --- | --- | --- | --- | --- | --- |
|  |  |  |  |  |  |  |
| Ctrl | 1.4±0.1 | 1.6±0.1 | 90.0 | 18.9±0.1 | 12.3±0.8 | 1.70±0.02 |
| PIRS | 1.4±0.1 | 1.7±0.1 | 89.3 | 19.0±0.2 | 12.9±1.0 | 1.71±0.03 |

All the values did not differ significantly (P>0.05) between PIRS and controls. In the food/water intake experiments, each treatment was repeated 6 times with each replicate containing one mouse. In the pregnancy outcome experiments, each treatment was repeated 3 times with each replicate containing about 10 mice.


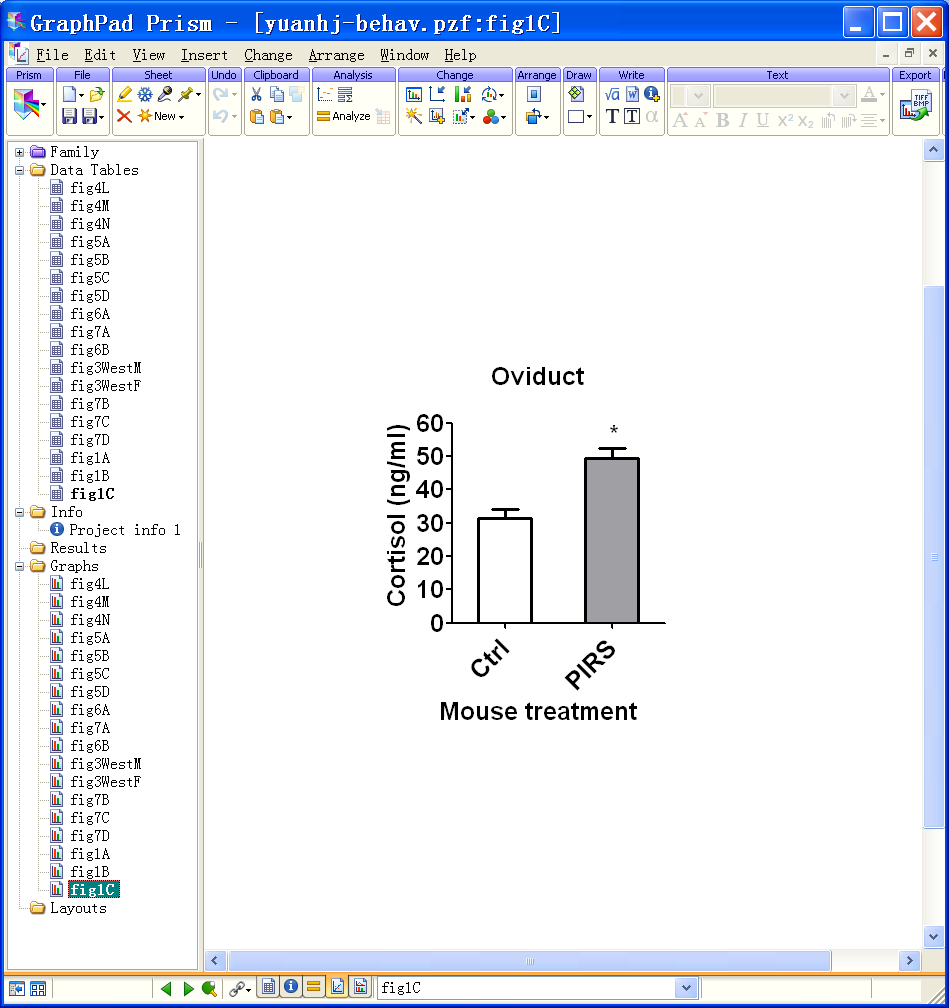

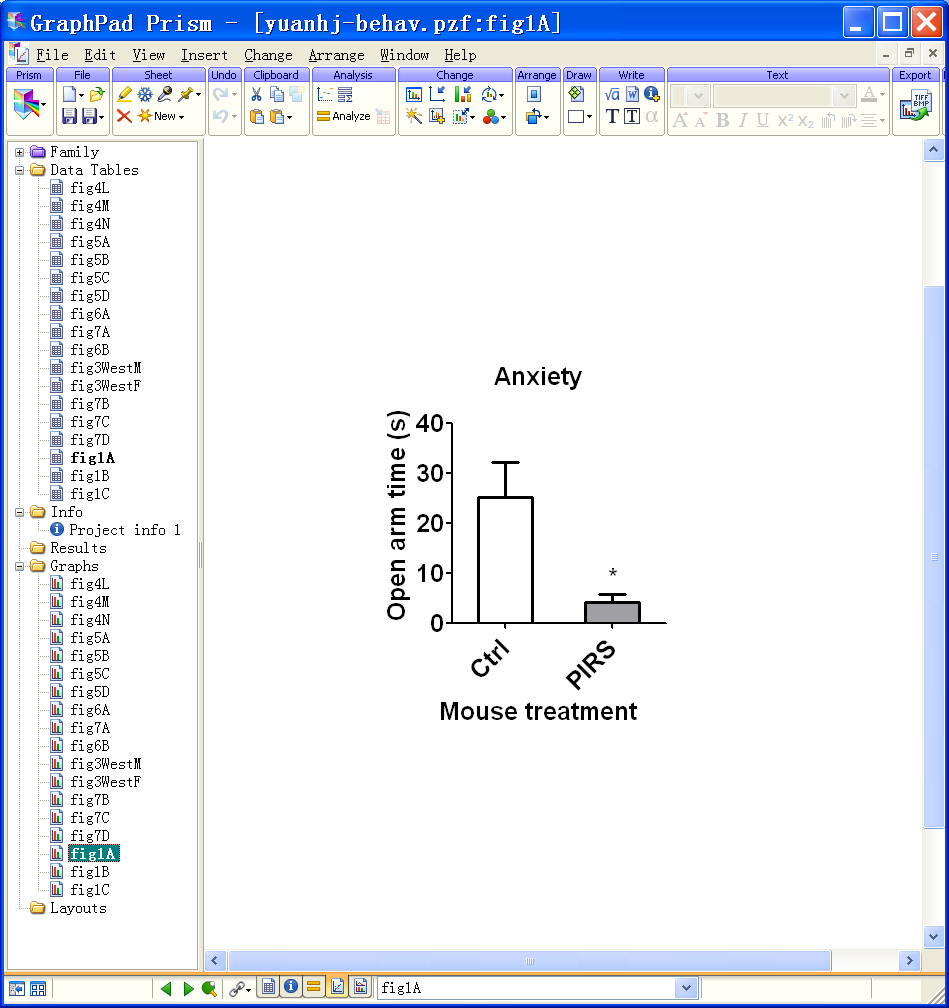

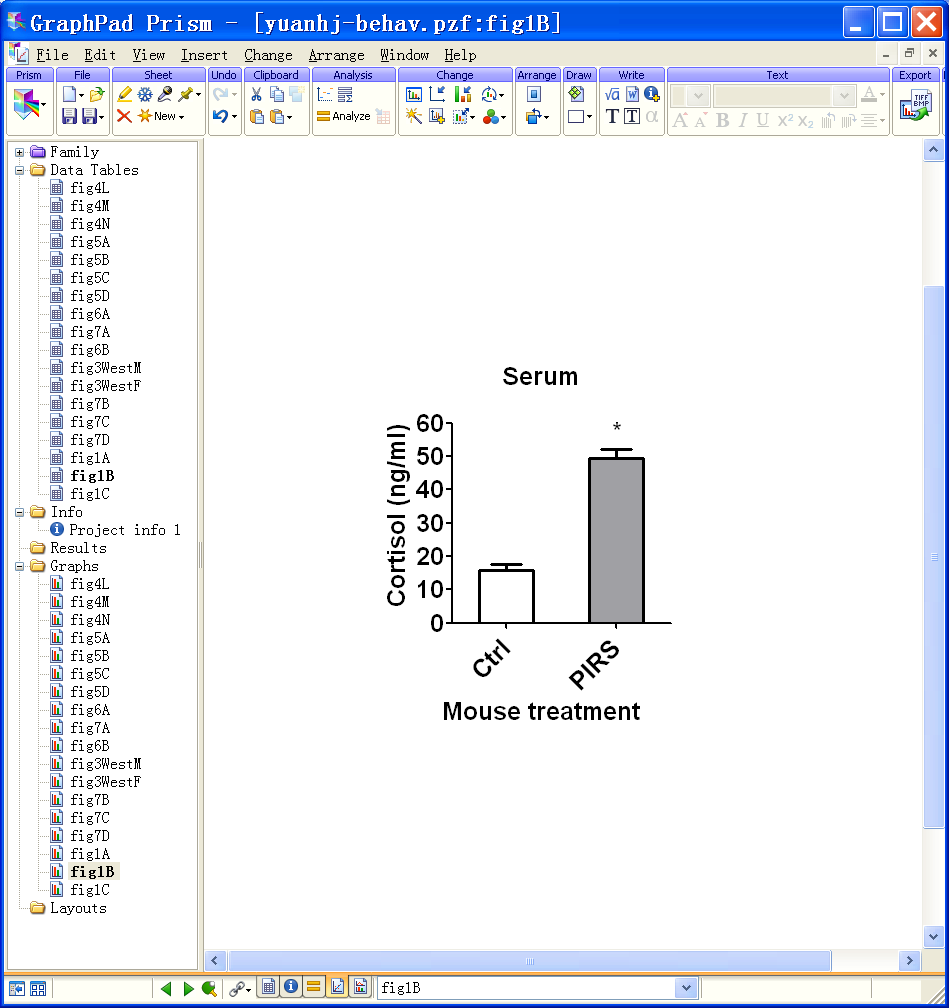


Figure S1. Open arm times of EPM and cortisol concentrations in serum or oviducts in pregnant mice after PIRS. For behavior test, each treatment was repeated 3 times with each replicate containing 5 mice. For cortisol measurement, each treatment was repeated 6-9 times with each replicate containing one mouse. *indicates significant difference (P<0.05) between PIRS and control (Ctrl) mice.


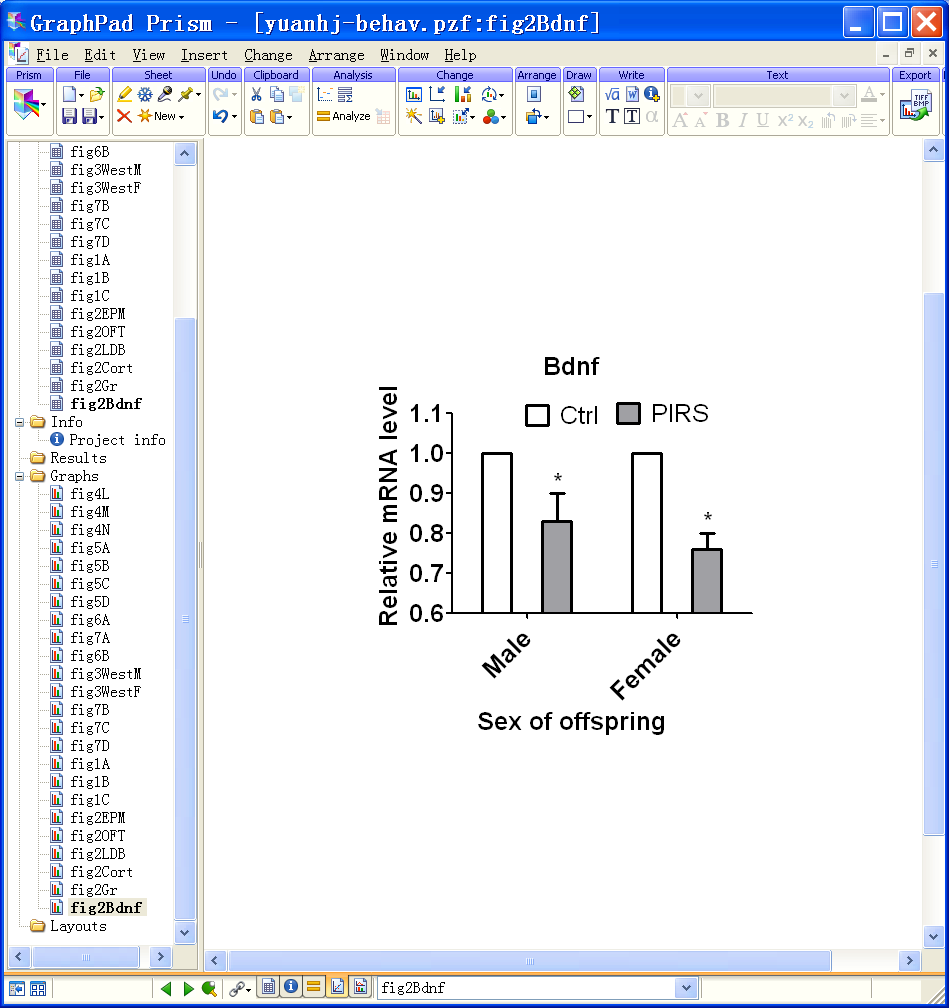

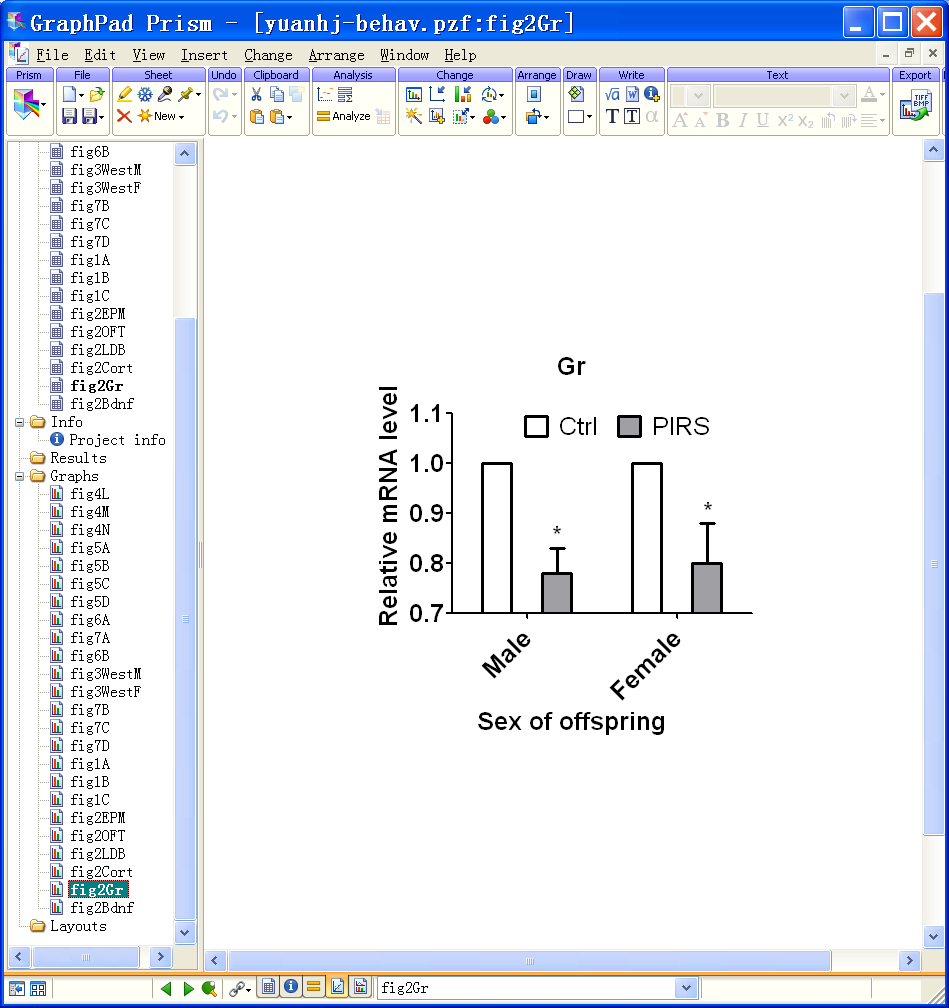

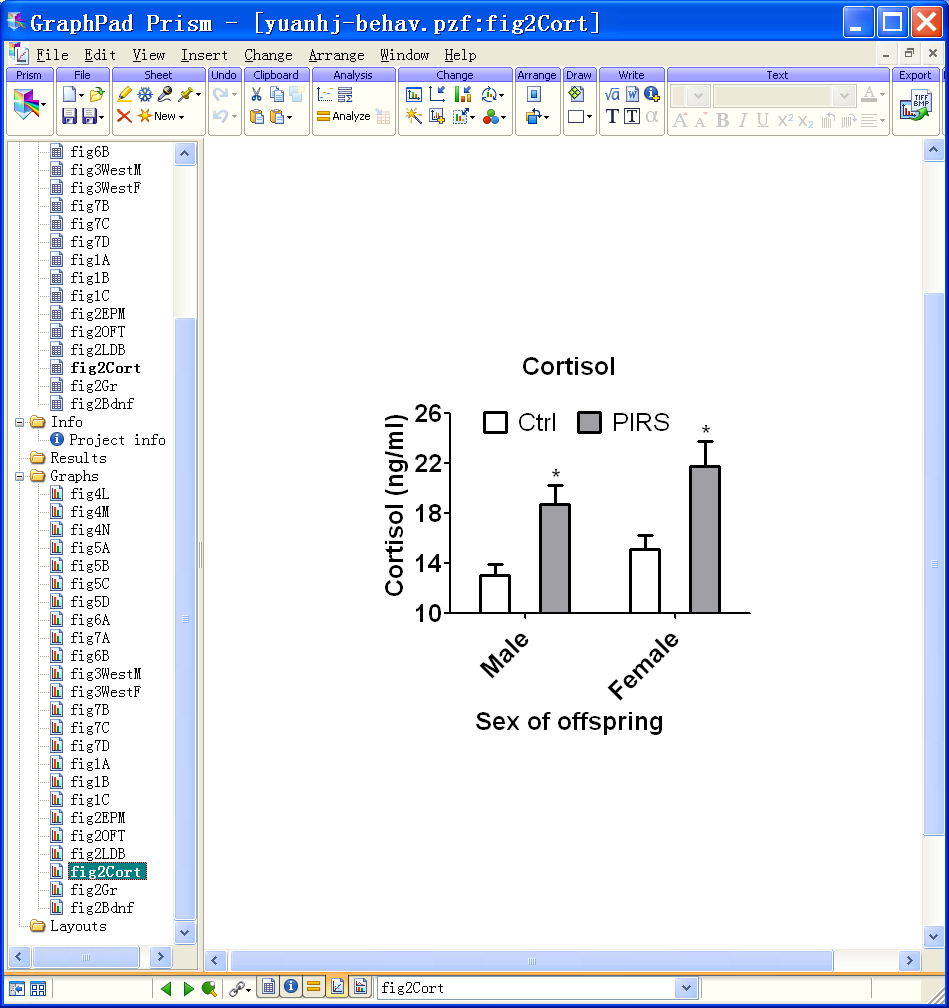

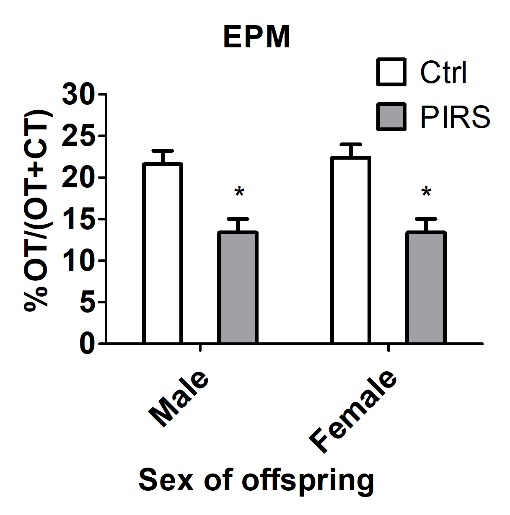

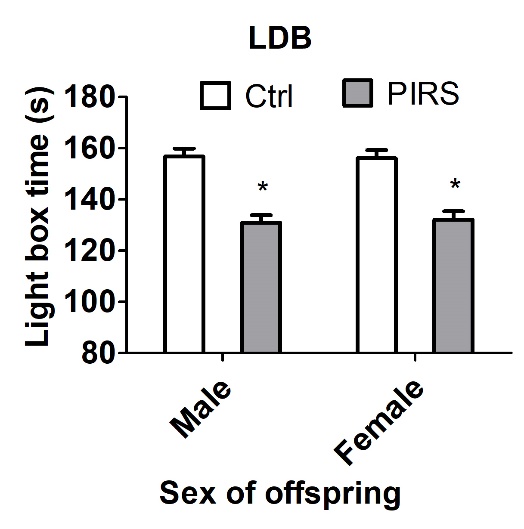

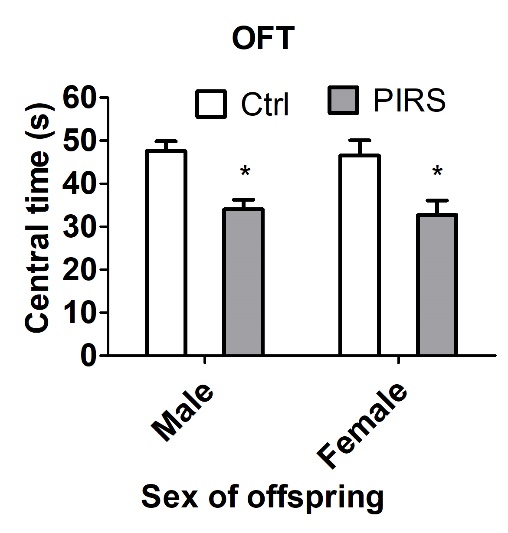


Figure S2. ALB, serum cortisol concentration, and hippocampal levels of Gr and Bdnf mRNAs in offspring from control (Ctrl) or PIRS mothers. The ALB was measured by % Open-arm time (OT)/OT + closed arm time (CT) of EPM, and times (s) in central area of OFT and in light box of LDB test. For behavioral tests, each treatment was repeated 3 times and each replicate contained about 20 offspring from 6-8 mother mice. For cortisol measurement, each treatment was repeated 3 times with each replicate containing 4 serum samples from 4 offspring each from a different mother. For RT-PCR analysis of Gr and Bdnf mRNAs, each treatment was repeated 3 times with each replicate containing 3 hippocampus samples from 3 offspring each from a different mother. * indicates significant difference (P<0.05) between Ctrl and PIRS within male or female offspring groups.

18.0% (14)


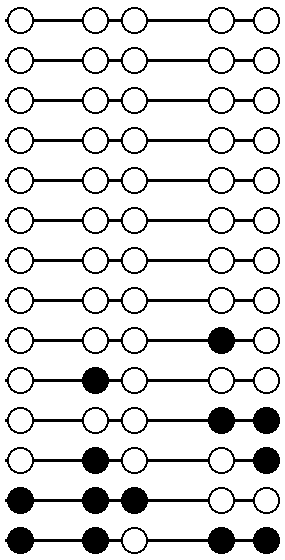

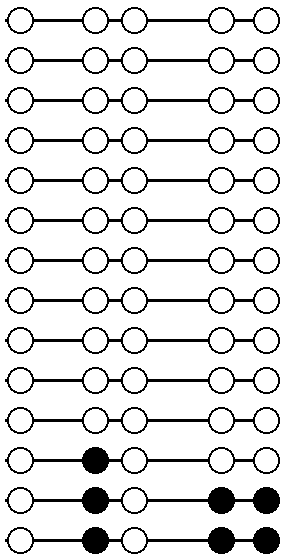


9.5% (14)

**MF**

**Ctrl**

**PIRS**


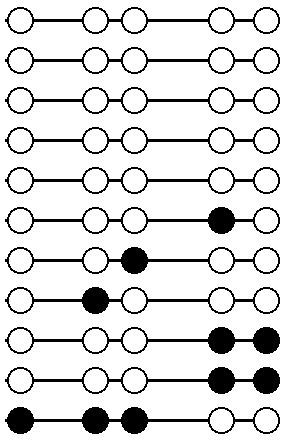

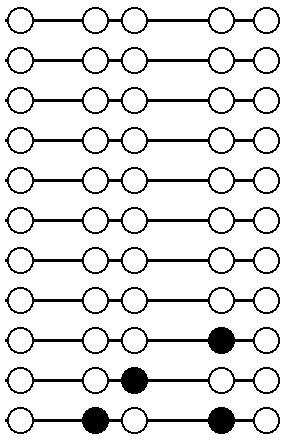


18.8% (11)

8.7% (11)

**FF**

**Ctrl**

**PIRS**


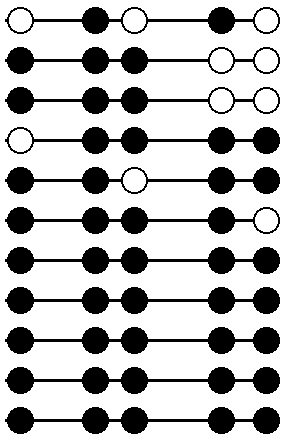

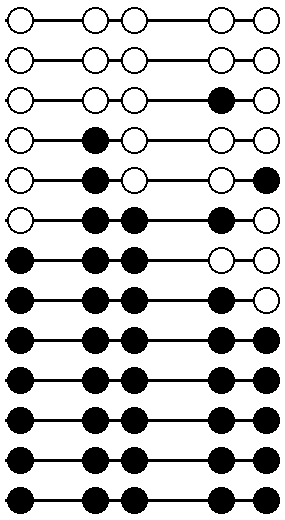


74.5% (11)

62.6% (13)

**Ctrl**

**PIRS**

**MA**


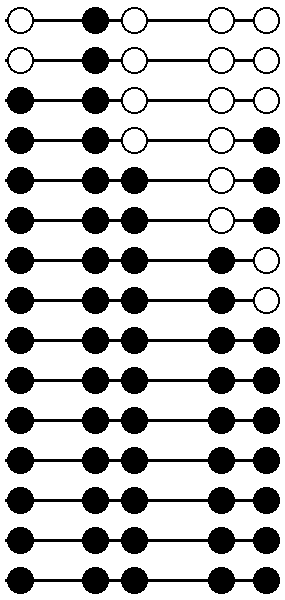

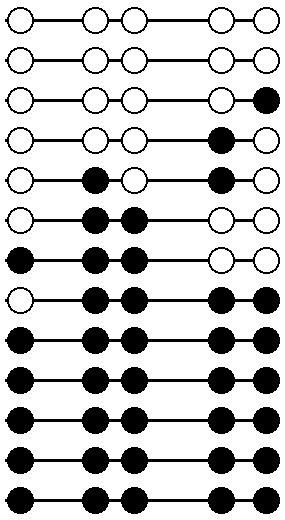


73.3% (15)

59.5% (13)

**FA**

**PIRS**

**Ctrl**

**C**


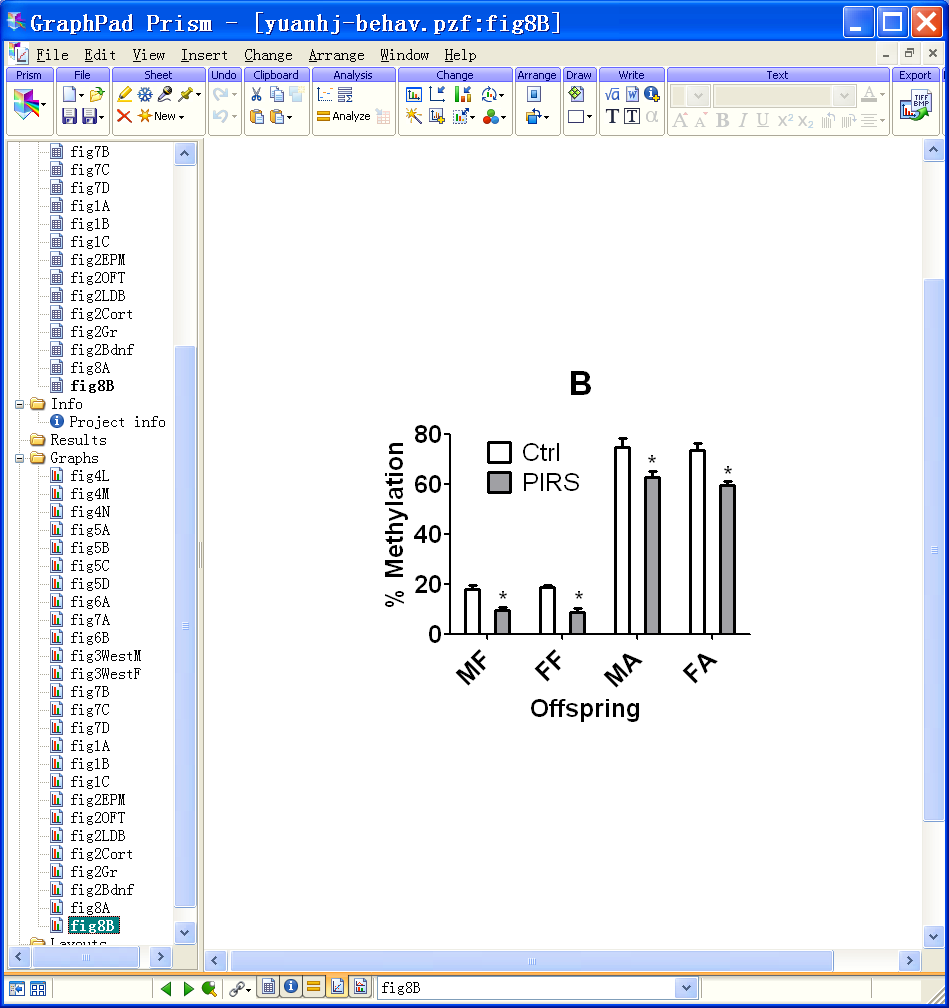

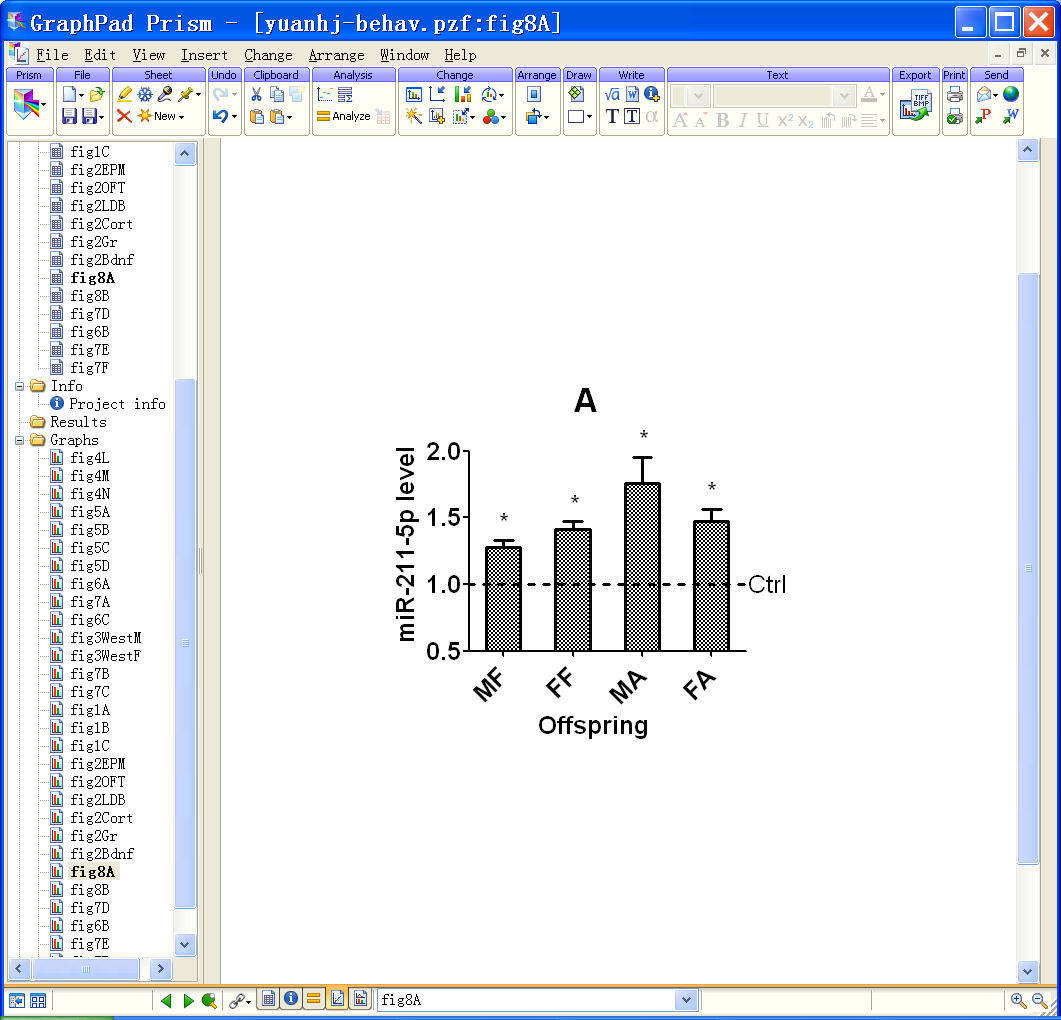


Figure S3. miR-211 expression and Trpm1 promoter methylation in hippocampi of fetal and adult offspring following maternal PIRS. Graph A shows miR-211-5p levels measured by quantitative real-time PCR in hippocampi of male (M) and female (F) fetal (F) or adult (A) offspring from PIRS mothers. The miR-211 level was calculated relative to that in offspring from unstressed control (Ctrl) mothers, which was set to one (dotted line). Each treatment was repeated 6 times with each replicate containing hippocampi from one animal from a different litter. Graph B shows percentages of methylated CpGs of the Trpm1 gene measured by bisulfate sequencing in hippocampi from MF, FF, MA and FA offspring derived from PIRS or control mothers. Each treatment was repeated 3 times with each replicate containing hippocampi from one fetus or adult from a different litter. * indicates a significant difference (P<0.05) from control offspring. Panel C shows DNA bisulfite sequencing of the Trpm1 gene in hippocampi of MF, FF, MA and FA offspring from PIRS or Ctrl mothers. White circles indicate unmethylated CpGs, and black circles represent methylated CpGs. Values on each treatment indicate the percentage of CpG methylation, and the number in parentheses indicates clones analyzed.
